# Preoperative magnetic resonance imaging criteria for predicting lymph node metastasis in patients with stage IB1‐IIA2 cervical cancer

**DOI:** 10.1002/cam4.4075

**Published:** 2021-07-18

**Authors:** Fangjie He, Shuiling Zu, Xia Chen, Jianping Liu, Ying Yi, Haijun Yang, Fuqiang Wang, Songhua Yuan

**Affiliations:** ^1^ Department of Obstetrics and Gynecology The First People's Hospital of Foshan Foshan China; ^2^ Nursing Department The Third Affiliated People’s Hospital of Fujian University of Traditional Chinese Medicine Fuzhou China; ^3^ Department of Radiology The First People's Hospital of Foshan Foshan China; ^4^ Department of Pathology The Anyang Tumor Hospital Anyang China

**Keywords:** adjuvant therapy, cervical cancer, lymph node, magnetic resonance imaging, tumor diameter

## Abstract

**Objective:**

This study aimed to identify patients with stage IB1‐IIA2 cervical cancer at low risk for lymph node metastasis (LNM) using preoperative magnetic resonance imaging (MRI) parameters.

**Methods:**

Clinical and MRI data of patients with stage IB1‐IIA2 cervical cancer who underwent radical surgery between 2010 and 2015 were retrospectively reviewed. Clinical stage IB1‐IIA2 cervical cancer was diagnosed according to the 2009 International Federation of Gynecology and Obstetrics staging system. The low‐risk criteria for LNM were identified using logistic regression analysis. The performance of the logistic regression analysis was estimated through receiver operating characteristic curve analysis.

**Results:**

Of 453 patients, 105 (23.2%) exhibited pathological LNM (p‐LNM). The maximal tumor diameter (adjusted odds ratio [aOR], 1.586; 95% confidence interval [CI], 1.312–1.916; *p *< 0.001) and LNM (aOR, 2.384; 95% CI, 1.418–4.007; *p* = 0.001) on preoperative MRI (m‐LNM) were identified as independent risk factors for p‐LNM using a multivariate logistic analysis. The p‐LNM rate was 4.0% for low‐risk patients (n = 124) identified using the current criteria (maximal tumor diameter <3.0 cm and no sign of m‐LNM). The 5‐year disease‐free survival rate of low‐risk patients was significantly greater than the rate of patients with a maximal tumor diameter ˃3.0 cm and/or signs of m‐LNM (90.4% vs. 82.1%; *p* = 0.033).

**Conclusions:**

The low‐risk criteria for p‐LNM were a maximal tumor diameter <3.0 cm and no sign of m‐LNM. Patients with stage IB1‐IIA2 cervical cancer at low risk for m‐LNM could be candidates for radical surgery; hence, they have a lesser need for adjuvant chemoradiotherapy, thus avoiding the severe comorbidities it causes.

## Graphical abstract

There was extremely low proportion of lymph node metastasis in patients categorized as low risk based on our criterion (5/124). Low risk is defined as maximal tumor diameter ≤3.0 cm and no indication of lymph node metastasis in MRI. Patients in the low risk could be identified for less adjuvant chemoradiotherapy and severe comorbidities.

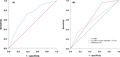

## 1 INTRODUCTION

Cervical cancer is the fourth most common cancer among women worldwide; in 2018, there were approximately 570,000 new cervical cancer cases and 311,000 deaths worldwide.^1^ Radical hysterectomy and pelvic lymphadenectomy have been proven to be effective and provide a satisfactory survival rate for patients with stage IB1‐IIA2 cervical cancer.^2^ Approximately 8%–26% of patients who have undergone lymphadenectomy exhibit pathological lymph node metastasis (p‐LNM).^3^ Among these patients, adjuvant chemoradiotherapy should be administered to decrease the incidence of tumor recurrence and improve survival.^2^ However, the combination of radical surgery and adjuvant chemoradiotherapy is associated with severe comorbidities, including lymphedema, lymphocyst, gastrointestinal morbidities, and genitourinary complications.^4^, ^5^ Hence, surgery is suitable for patients with early‐stage cervical cancer without p‐LNM. Therefore, identification of the low‐risk group is of interest.

For patients with limited tumor diameter, stromal invasion, lymphovascular space invasion, parametrial involvement, or uterine corpus invasion, the rate of p‐LNM could be significantly decreased^6^, ^7^, ^8^; however, these parameters, except for tumor diameter, were obtained from postoperative pathological examinations. Although preoperative evaluations of lymph node status are limited, preoperative magnetic resonance imaging (MRI) could potentially be used to evaluate the risk of p‐LNM and to perform accurate and reproducible assessments of the tumor diameter.^9^, ^10^, ^11^, ^12^ The reported sensitivity and specificity rates for identifying p‐LNM through MRI are 17.2%–59.1% and 78.8%–99.1%, respectively.^9^, ^10^, ^11^, ^12^ This revealed that MRI has a low sensitivity for detecting p‐LNM, which means a considerably high missed diagnosis rate.

This study aimed to identify the low‐risk group of p‐LNM patients among individuals with stage IB1‐IIA2 cervical cancer using preoperative MRI parameters.

## 2 PATIENTS AND METHODS

### 2.1 Patients

This retrospective study was approved by the Institutional Review Board of the First People's Hospital of Foshan (L2020‐17); informed consent from patients was waived. The study was conducted in accordance with the Declaration of Helsinki of 1964 and its subsequent revisions.

Medical records and MRI data of patients who were surgically treated between 2010 and 2015 at our institution were collected and reviewed. The inclusion criteria were as follows: diagnosis of clinical stage IB1‐IIA2 cervical cancer according to the 2009 International Federation of Gynecology and Obstetrics (FIGO) staging system^13^; presence of squamous cell carcinoma, adenocarcinoma, or adenosquamous carcinoma; history of type C radical hysterectomy and bilateral pelvic lymphadenectomy; MRI <14 days before surgery. The exclusion criteria were as follows: adjuvant chemotherapy or radiotherapy before surgery, conization before MRI, and poor image quality due to imaging artifacts.

A total of 453 patients were included. Patients were divided into two groups according to their postoperative pathological lymph node status: p‐LNM group (with LNM; n = 105) and non‐p‐LNM group (without LNM; n = 348).

### 2.2 MRI protocols

Each patient underwent preoperative MRI using a 1.5‐T system with a pelvic array coil (Intera; Philips Medical System, Best, Netherlands). The following parameters were applied for the axial T1‐weighted fast spin‐echo (FSE) sequence: repetition time (TR)/echo time (TE), 600 ms/10 ms; slice thickness, 5 mm; interslice gap, 2 mm; field of view (FOV), 24 × 24 cm; matrix, 256 × 192; echo train length (ETL), 4; number of excitations (NEX), 3; bandwidth, 31.25 kHz. For the axial T2‐weighted FSE sequence, the following parameters were applied: TR/TE, 5,000 ms/68 ms; slice thickness, 3 mm; interslice gap, 1 mm; FOV, 24 × 24 cm; matrix, 256 × 192; ETL, 21; NEX, 4; bandwidth, 31.25 kHz. For the sagittal and coronal T2‐weighted FSE sequence, the following parameters were applied: TR/TE, 5,000 ms/68 ms; slice thickness, 3 mm; interslice gap, 3 mm; FOV, 24 × 24 cm; matrix, 256 × 192; ETL, 26; NEX, 4; bandwidth, 31.25 kHz. For the gadolinium‐enhanced axial and sagittal T1‐weighted turbo spin‐echo sequence for the pelvic region, the following parameters were applied: TR/TE, 175 ms/4.2 ms; slice thickness, 5 mm; interslice gap, 2 mm; FOV for the axial and sagittal plane, 24 × 24 cm; matrix, 256 × 192; ETL, 3; NEX, 4; bandwidth, 31.25 kHz. For the axial T2‐weighted FSE sequence with 16 s of breath‐holding for the para‐aortic region, the following parameters were applied: TR/TE, 2,000 ms/68 ms; slice thickness, 8 mm; interslice gap, 2 mm; FOV for the axial and sagittal plane, 32 × 24 cm; matrix, 256 × 160; ETL, 20; NEX, 1; bandwidth, 31.25 kHz.

### 2.3 Image analysis

Two radiologists blinded to the surgical results reviewed the preoperative MRI results and determined LNM (m‐LNM) according to whether the short‐axis diameter of the largest lymph node was ≥10 mm based on gadolinium‐enhanced axial and sagittal T1‐weighted turbo spin‐echo sequence.^14^ Lymph nodes were classified according to the name of the adjacent vessel, including para‐aortic areas, both common iliac areas, both external iliac areas, and both internal iliac/obturator areas. The largest short‐axis diameter of the largest lymph node in the above areas was measured.^11^ The maximal tumor diameters were determined based on axial, sagittal, and coronal T2‐weighted images. Any disagreement was resolved by consultation.

### 2.4 Pathological examination and end point

A retrospective review of hematoxylin and eosin‐stained lymph node tissue specimens was independently performed by two pathologists who were blinded to patient outcomes. The results of lymph node tissue re‐examination were consistent with those in the original pathological reports. Moreover, pelvic and para‐aortic (in some patients) lymph nodes were evaluated. Surgical specimens of the left and right pelvic lymph nodes were not divided into detailed sites for pathological examination because they would not influence the postoperative treatment strategy.

Patients were followed up until September 30, 2020, through an outpatient information inquiry and examination report system. The primary end point was lymph node status, which was determined through pathological examination after surgery. The secondary end point was 5‐year disease‐free survival (DFS), which was calculated as the number of months from the date of diagnosis to either the first evidence of recurrence or death from cervical cancer.

### 2.5 Statistical analysis

Student's t‐test and chi‐squared test were used to compare the continuous and categorical variables of the two groups, respectively. A multivariate, forward stepwise logistic regression analysis was performed to identify the independent risk factors for p‐LNM. The criteria and cutoff values for p‐LNM prediction were determined through a receiver operating characteristic (ROC) curve analysis. The 5‐year DFS was estimated using the Kaplan–Meier method; differences between groups were compared using the log‐rank test. All statistical analyses were performed using SPSS version 26.0 (IBM Inc.) and STATA version 15.0 (College Station, TX, USA). Two‐sided *p* < 0.05 was considered statistically significant.

## 3 RESULTS

### 3.1 Patient characteristics

Patient selection is shown in Figure S1, and the clinical and pathological characteristics of all patients are summarized in Table S1. Of 453 patients, 105 (23.2%) had pathology results indicating p‐LNM and 91 (20.1%) had preoperative MRI results indicating m‐LNM. Of the 3,171 lymph node regions (seven regions per patient), 194 (6.1%) had metastasis based on the preoperative MRI results (Table S2).

The clinical characteristics and preoperative MRI results of the patients with and without p‐LNM were compared (Table 1). Compared with patients without p‐LNM, patients with p‐LNM had significantly advanced cancer (*p* = 0.033), had significantly larger tumor diameters (*p* < 0.001), and were more likely to exhibit uterine corpus invasion and m‐LNM on preoperative MRI.

**TABLE 1:** Clinical characteristics and MRI findings of patients with p‐LNM or without p‐LNM

Characteristic	p‐LNM	Without p‐LNM	*p*
Number (%)	105 (23.2)	348 (76.8)	
Age, mean (SD), years	48.9 (9.7)	50.0 (9.3)	0.302
Stage			**0.033**
IB1	45 (42.9)	194 (55.7)	
IB2	19 (18.1)	35 (10.1)	
IIA1	27 (25.7)	89 (25.6)	
IIA2	14 (13.3)	30 (8.6)	
Histological type, n (%)			0.290
Squamous cell carcinoma	83 (79.0)	296 (85.1)	
Adenocarcinoma	17 (16.2)	43 (12.4)	
Adenosquamous carcinoma	5 (4.8)	9 (2.6)	
Grade, n (%)			0.191
G1	4 (3.8)	19 (5.5)	
G2	37 (35.2)	154 (44.3)	
G3	60 (57.1)	169 (48.6)	
Unknown	4 (3.8)	6 (1.7)	
Preoperative MRI findings			
Tumor diameter, mean (SD), cm	4.0 (1.2)	3.2 (1.2)	**<0.001**
PMI, n (%)			0.354
No	100 (95.2)	338 (97.1)	
Yes	5 (4.8)	10 (2.9)	
Uterine corpus invasion			**0.001**
No	88 (83.8)	328 (94.3)	
Yes	17 (16.2)	20 (5.7)	
m‐LNM, n (%)			**<0.001**
No	68 (64.8)	294 (84.5)	
Yes	37 (35.2)	54 (15.5)	

Bold values means *p* values less than 0.05.

Abbreviations: m‐LNM, lymph node metastasis on preoperative MRI; MRI, magnetic resonance imaging; p‐LNM, pathological lymph node metastasis; PMI, parametrial involvement; SD, standard deviation.

### 3.2 Risk factors for P‐LNM

The risk factors for p‐LNM according to the univariate and multivariate binary logistic regression analyses of the preoperative clinical characteristics and MRI findings are shown in Table 2. In the univariate analysis, the grades and all preoperative MRI parameters, except for parametrial involvement, were significantly associated with p‐LNM. In the multivariate analysis, tumor diameter and m‐LNM on preoperative MRI were identified as independent risk factors for p‐LNM.

**TABLE 2:** Univariate and multivariate analyses of risk factors for p‐LNM by binary logistic regression models

Risk factors	Univariate analysis		Multivariate analysis	
	OR (95% CI)	*p*	aOR (95% CI)	*p*
Age	0.988 (0.965–1.011)	0.301		0.776
Stage	1.206 (0.990–1.470)	0.063		0.977
Histologic type	1.409 (0.916–2.167)	0.119		0.059
Grade	1.209 (1.001–1.460)	0.049		0.082
Preoperative MRI findings				
Tumor diameter	1.666 (1.385–2.003)	< 0.001	1.586 (1.312–1.916)	**<0.001**
PMI	1.690 (0.565–5.059)	0.348		0.474
Uterine corpus invasion	3.168 (1.592–6.304)	0.001		0.185
m‐LNM	2.962 (1.807–4.857)	< 0.001	2.384 (1.418–4.007)	**0.001**

None of the listed covariates had multicollinearity. The Hosmer and Lemeshow test (chi‐squared value = 5.824; *p* = 0.560) for binary multivariate logistic regression was used.

Bold values means *p* values less than 0.05.

Abbreviations: aOR, adjusted odds ratio; CI, confidence interval; m‐LNM, lymph node metastasis on preoperative MRI; MRI, magnetic resonance imaging; OR, odds ratio; p‐LNM, pathological lymph node metastasis; PMI, parametrial involvement.

### 3.3 Cutoff values of the tumor diameter

**FIGURE 1:**
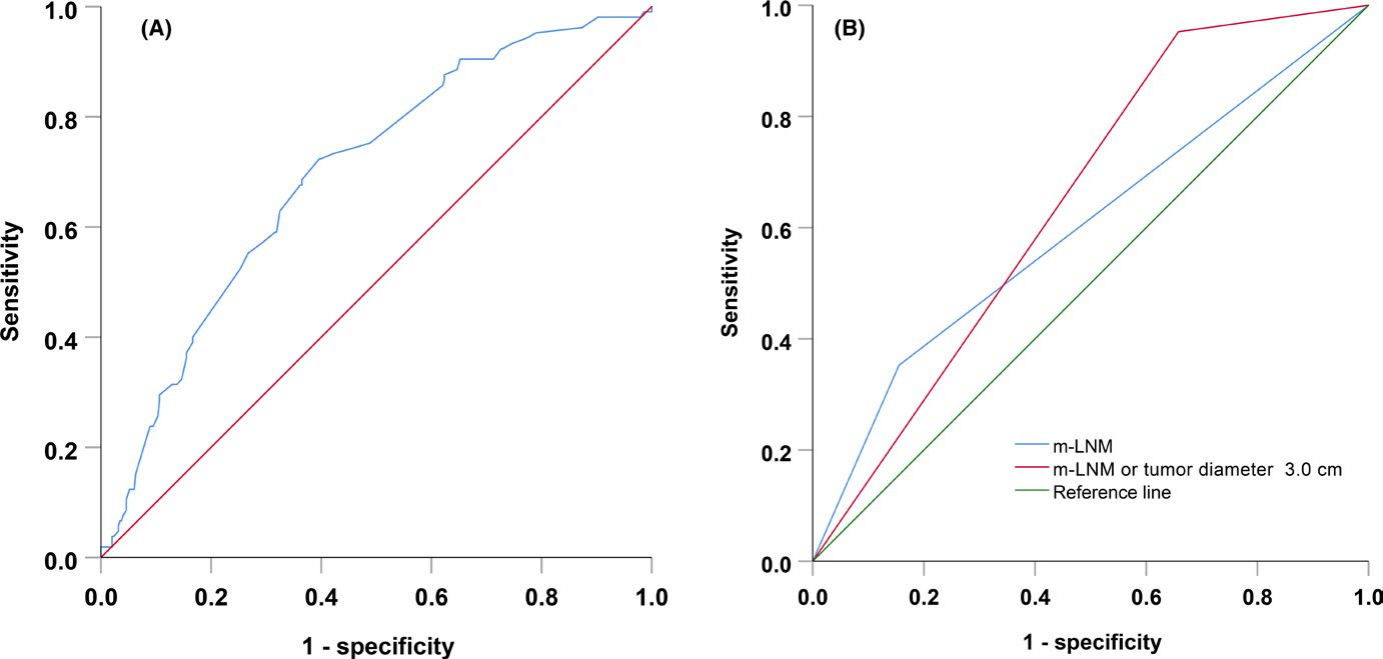
Receiver operating characteristic curves for pathological lymph node metastasis prediction: (A) tumor diameter and (B) predictive performance

The optimal cutoff value for tumor diameter was determined using a ROC curve analysis and was calculated as 3.0 cm, which had optimal sensitivity and specificity (area under the ROC curve, 0.695; 95% confidence interval [CI], 0.640–0.751; *p* < 0.001) (Figure 1A).

### 3.4 Group at low risk for P‐LNM

The predictive performance of preoperative MRI and the MRI results indicating the tumor diameter and presence of m‐LNM are shown in Table 3. The two parameters (tumor diameter and presence of m‐LNM) were combined to improve the sensitivity and decrease missed diagnosis rate. Therefore, according to the preoperative MRI results, the group at low risk for p‐LNM was defined as having a tumor diameter <3.0 cm and no m‐LNM (Figure S2). The predictive model performance and preoperative MRI results for p‐LNM were compared (Figure 1B, Table 3). The specificity and positive predictive value of the model were lower, but the sensitivity and negative predictive value were higher than those for m‐LNM alone. A total of 124 patients (27.4%) were assigned to the low‐risk group; remarkably, only 5 patients (4.0%) exhibited p‐LNM. The predictive performance of pathological risk factors and adjuvant radiotherapy for the low‐risk group is shown in Table 4.

**TABLE 3:** Predictive model performance and preoperative MRI for p‐LNM

MRI findings	AUC, 95% CI	Sensitivity, %	Specificity, %	PPV, %	NPV, %
m‐LNM	0.599	35.2	84.5	40.7	81.2
m‐LNM or tumor diameter ≥3.0 cm	0.647	95.2	34.2	30.4	96.0

Abbreviations: AUC, area under the receiver operating characteristic curve; m‐LNM, lymph node metastasis on preoperative MRI; MRI, magnetic resonance imaging; NPV, negative predictive value; p‐LNM, pathological lymph node metastasis; PPV, positive predictive value.

**TABLE 4:** Predictive performance of pathological risk factors and adjuvant radiotherapy for the low‐risk group

Risk factors	Low‐risk group	Without m‐LNM
Number (%)	124 (100.0)	362 (100.0)
Tumor diameter ≥4 cm	3 (2.4)	66 (18.2)
Stromal invasion depth ˃1/2	56 (45.2)	244 (67.4)
LVSI	32 (25.8)	101 (27.9)
PMI	2 (1.6)	8 (2.2)
RMI	3 (2.4)	11 (3.0)
p‐LNM	5 (4.0)	68 (18.8)
Adjuvant radiotherapy	28 (22.6)	154 (42.5)

Abbreviations: LVSI, lymphovascular space invasion; m‐LNM, lymph node metastasis on preoperative MRI; MRI, magnetic resonance imaging; p‐LNM, pathological lymph node metastasis; PMI, parametrial involvement; RMI, resection margin involvement.

### 3.5 Survival analysis

**FIGURE 2:**
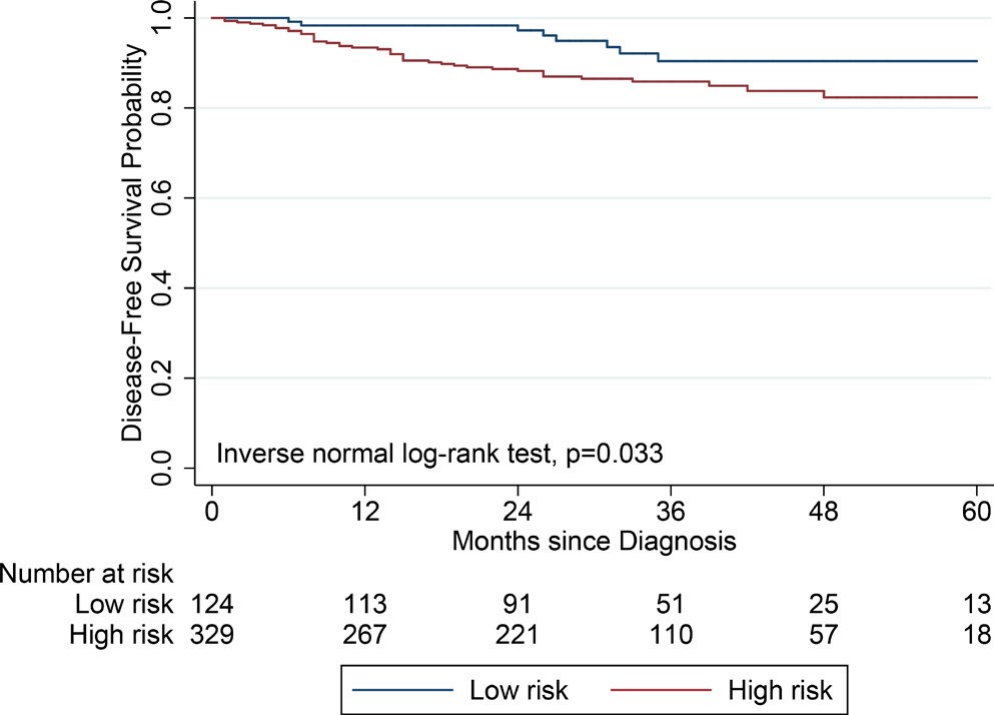
Secondary outcomes: 5‐year disease‐free survival according to the predictive performance for p‐LNM. Low risk: tumor diameter <3.0 cm and no sign of m‐LNM. High‐risk: tumor diameter ≥3.0 cm and/or sign of m‐LNM. p‐LMN, pathological lymph node metastasis; m‐LNM, lymph node metastasis on preoperative magnetic resonance imaging

The median follow‐up duration for all patients was 32 months; the 5‐year DFS was 90.4% of the low‐risk group and 82.1% (*p* = 0.033) for patients with a tumor diameter ≥3.0 cm and/or signs of m‐LNM (Figure 2). Additionally, the 5‐year DFS of the low‐risk group was higher than that of the group without signs of m‐LNM (85.1%).

## 4 DISCUSSION

This study revealed that patients with stage IB1‐IIA2 cervical cancer were at low risk for p‐LNM when they had a tumor diameter <3.0 cm and had no m‐LNM according to their preoperative MRI results. Furthermore, MRI was able to identify patients with stage IB1‐IIA2 cervical cancer at low risk for p‐LNM, thereby allowing for the potential to avoid several comorbidities and improve their quality of life.

Poor outcomes are associated with p‐LNM, and additional adjuvant radiotherapy with or without concurrent chemotherapy is required for patients with cervical cancer. According to the 2018 FIGO cervical cancer staging, LNM was assigned to stage IIIC^15^; therefore, computed tomography (CT) and MRI are recommended to help determine the tumor extension, lymph node, and staging.^15^, ^16^ Previous studies have revealed that CT and MRI have considerably high specificity (>85%) but low sensitivity (<65%) for detecting p‐LNM.^17^, ^18^, ^19^ Positron emission tomography‐CT is also used to detect p‐LNM in patients suspected of having distant metastasis; however, a meta‐analysis revealed that its sensitivity for detecting p‐LNM is only 82%.^20^ These imaging modalities have low sensitivity for detecting p‐LNM, with a missed diagnosis rate >20%; this may be due to their inability to detect lymphatic metastasis in nodes with normal sizes and shapes.^19^

Tumor diameter is considered a good prognostic indicator of p‐LNM.^6^ Several previous studies have demonstrated that MRI is the optimal imaging modality for estimating tumor diameter.^10^, ^11^, ^12^ Additionally, correlation with postoperative measurement in tumor size was higher for MRI than for pelvic examination in patients with cervical cancer.^18^ In the present study, the tumor diameter determined through MRI was considered in logistic regression analysis and was identified as an independent risk factor for p‐LNM. Furthermore, to improve the sensitivity for detecting p‐LNM, we combined tumor diameter and m‐LNM using MRI for those in the low‐risk group. Compared with a previous study using a tumor diameter <2.6 cm that revealed a p‐LNM rate of 1.3% to 14.3%,^10^, ^11^ the present study revealed a p‐LNM rate of only 4.0%. Additionally, this study broadens this criterion to 3.0 cm, thus extending the clinical application. In one of these previous studies, Kang et al. further revealed that sensitivity was increased to 83.3% when a tumor diameter <2.6 cm was combined with lymph node diameter (9 mm), but their sensitivity rate is significantly lower than ours.^11^ The sensitivity was not higher in their models because they used linear regression to construct the model and formula. Another study attempted to diagnose p‐LNM by combining the tumor diameter with the apparent diffusion coefficient of the tumor; however, the sensitivity did not improve significantly and was as low as 59.1%.^12^

Some studies have attempted to identify p‐LNM with the help of MRI‐based radiomics analyses. Their models revealed that the sensitivity for detecting p‐LNM ranged from 84.3% to 92.96%^21^, ^22^, ^23^; however, the sensitivity rates reported remained slightly lower than ours. This would have resulted in a considerably high missed diagnosis rate for p‐LNM. A previous study reported that the sensitivity performance of their model could be as high as 94.3% for the training cohort and 100.0% for the validation cohort^24^; however, the criteria in the present study are more practical and can be easily used in clinical practice.

Except for imaging examination, two previous studies have used tumor biomarkers to predict p‐LNM and demonstrated that serum squamous cell carcinoma antigen was independently associated with p‐LNM. Their predictive models revealed sensitivity and specificity of 53.8%–70.0% and 63.0%–83.9%, respectively.^25^, ^26^ Furthermore, the expressions of matrix metalloproteinase 7 and 9 by tissue immunohistochemistry were revealed to be associated with p‐LNM, and the sensitivity rates were 91.4% and 71.4%, respectively.^27^ However, the above non‐imaging methods had lower sensitivity rates and higher missed diagnosis rates for p‐LNM than our methods.

Patients in the low‐risk group had a very low incidence of p‐LNM according to the current criteria, which can be used to decrease the risk of false‐negative p‐LNM diagnoses and were determined using postoperative pathological findings. Furthermore, excluding p‐LNM, the low‐risk group had fewer pathological risk factors and lower rates of adjuvant therapy use. Finally, the survival analysis revealed higher DFS; therefore, compared with stage IB1 and stage IIA1 cervical cancer patients, those in the low‐risk group (especially young women) are more suitable for radical surgery because adjuvant therapy is associated with lower rate of p‐LNM and has less severe comorbidities.

This study had several limitations. First, given the retrospective nature of this study, selection bias was inherent. Second, although MRI is recommended as a preoperative evaluation method for patients with cervical cancer, it may not be available in regions with low resources. Third, the criteria were not validated by other institutions. Fourth, the surgical specimens of the left and right pelvic lymph nodes have not been divided into detailed sites for pathological examination. Hence, lymph node involvement detected by MRI could not be matched to the pathological surgical specimens. However, it would not influence the postoperative treatment strategy.

## 5 CONCLUSIONS

This study demonstrated that preoperative MRI could identify patients at low risk for p‐LNM. A maximal tumor diameter less than 3.0 cm and the absence of m‐LNM on preoperative MRI were identified as independent predictive factors for p‐LNM. These criteria are valuable for excluding p‐LNM and help in identifying potential candidates for radical surgery among patients with IB1‐IIA2 cervical cancer. The low‐risk criteria for p‐LNM were a maximal tumor diameter <3.0 cm and no sign of m‐LNM.

## CONFLICT OF INTEREST

The authors declare that the research was conducted in the absence of any commercial or financial relationships that could be construed as a potential conflict of interest.

## AUTHOR CONTRIBUTIONS

SY conceived and designed the study. FH participated in the design and draft of the manuscript. SZ and XC performed the data collection and analysis. JL and YY reviewed magnetic resonance imaging. HY and FW reviewed the pathological examination. All authors read and approved the final manuscript.

## Supporting information

Fig S1

Fig S2

Table S1‐S2

## Data Availability

The data that support the findings of this study are available from the corresponding author, Songhua Yuan, upon reasonable request.
