# Supplementary figures and images for: Preoperative magnetic resonance imaging criteria for predicting lymph node metastasis in patients with stage IB1‐IIA2 cervical cancer
Source: Cancer Med. 2021 Jul 18;10(16):5429–36. doi: 10.1002/cam4.4075 (PMC8366085; doi:10.1002/cam4.4075)

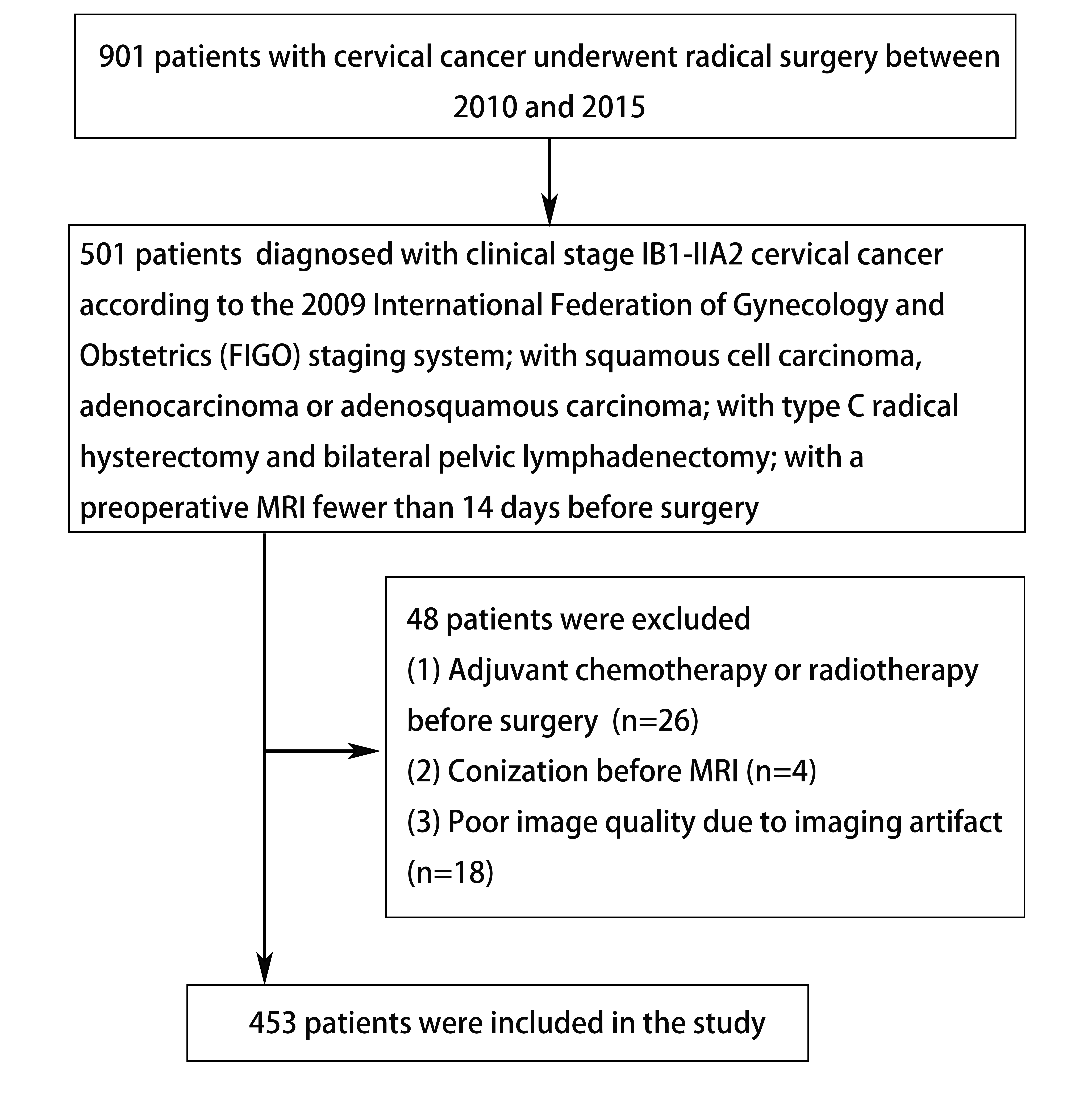

Supplement: Supplementary file 1 — Fig S1 [file CAM4-10-5429-s001.tif]

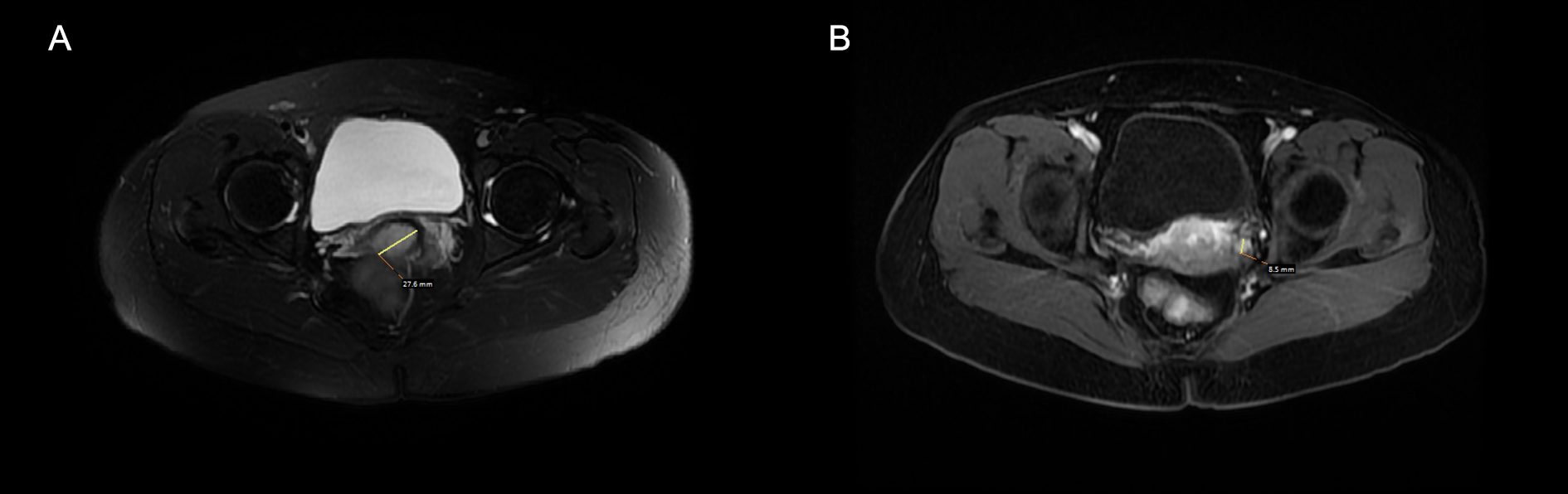

Supplement: Supplementary file 2 — Fig S2 [file CAM4-10-5429-s002.tif]
