# Supplementary material for: Preoperative magnetic resonance imaging criteria for predicting lymph node metastasis in patients with stage IB1‐IIA2 cervical cancer
Source: Cancer Med. 2021 Jul 18;10(16):5429–36. doi: 10.1002/cam4.4075 (PMC8366085; doi:10.1002/cam4.4075)
Supplement: Supplementary file 3 — Table S1‐S2 [file CAM4-10-5429-s003.docx]

Supplementary Material

# Supplementary Tables

**Supplementary Table 1**. Clinical and pathological characteristics of 453 cervical cancer patients

| Characteristic | N (%) |
| --- | --- |
| Age, mean (range), years | 49.7 (25–73) |
| Stage |  |
| IB1 | 239 (52.8) |
| IB2 | 54 (11.9) |
| IIA1 | 116 (25.6) |
| IIA2 | 44 (9.7) |
| Histological type, n (%) |  |
| Squamous cell carcinoma | 379 (83.7) |
| Adenocarcinoma | 60 (13.2) |
| Adenosquamous carcinoma | 14 (3.1) |
| Grade, n (%) |  |
| G1 | 23 (5.1) |
| G2 | 191 (42.2) |
| G3 | 229 (50.6) |
| Unknown | 10 (2.2) |
| Preoperative MRI findings |  |
| Tumor diameter, mean (SD), cm | 3.4 (1.2) |
| PMI, n (%) |  |
| No | 438 (96.7) |
| Yes | 15 (3.3) |
| Uterine corpus invasion |  |
| No | 416 (91.8) |
| Yes | 37 (8.2) |
| m-LNM, n (%) |  |
| No | 362 (79.9) |
| Yes | 91 (20.1) |
| Pathological findings |  |
| Tumor diameter, mean (SD), cm | 3.2 (1.3) |
| Stromal invasion depth ˃1/2, n (%) |  |
| No | 145 (32.0) |
| Yes | 308 (68.0) |
| LVSI, n (%) |  |
| No | 322 (71.1) |
| Yes | 131 (28.9) |
| PMI, n (%) |  |
| No | 439 (96.9) |
| Yes | 14 (3.1) |
| RMI, n (%) |  |
| No | 439 (96.9) |
| Yes | 14 (3.1) |
| p-LNM, n (%) |  |
| No | 348 (76.8) |
| Yes | 105 (23.2) |
| Adjuvant radiotherapy, n (%) |  |
| No | 243 (53.6) |
| Yes | 210 (46.4) |

MRI, magnetic resonance imaging; PMI, parametrial involvement; m-LNM, lymph node metastasis on preoperative MRI; LVSI, lymphovascular space invasion; RMI, resection margin involvement; p-LNM, pathological lymph node metastasis.

**Supplementary Table 2**. Details of positive lymph node metastasis (seven regions) based on MRI of 453 cervical cancer patients

| Characteristic | N (%) |
| --- | --- |
| Para-aortic lymph nodes, n (%) | 2 (0.4) |
| Common iliac lymph nodes, n (%) |  |
| Left | 31 (6.8) |
| Right | 29 (6.4) |
| External iliac lymph nodes, n (%) |  |
| Left | 20 (4.4) |
| Right | 19 (4.2) |
| Internal iliac/obturator lymph nodes, n (%) |  |
| Left | 51 (11.3) |
| Right | 42 (9.3) |

MRI, magnetic resonance imaging; LNM, lymph node metastasis.

## Supplementary Figures

**Supplementary Figure 1.** Flowchart of patient selection

**Supplementary Figure 2.** Low-risk patients with cervical cancer based on MRI image. (A) The tumor diameter is <3.0 cm, based on axial, sagittal, and coronal T2-weighted images; (B) the short-axis diameter of the largest lymph node is <10 mm based on gadolinium-enhanced axial and sagittal T1-weighted turbo-spin echo sequence.
